# Identification of novel mutations among Iranian NPC1 patients: a bioinformatics approach to predict pathogenic mutations

**DOI:** 10.1186/s41065-022-00224-1

**Published:** 2022-01-27

**Authors:** Rezvan Abtahi, Parvaneh Karimzadeh, Omid Aryani, Diba Akbarzadeh, Shadab Salehpour, Alireza Rezayi, Seyed Hassan Tonekaboni, Reza Zolfaghari Emameh, Massoud Houshmand

**Affiliations:** 1 Department of Medical Genetics, National Institute for Genetic Engineering and Biotechnology, (NIGEB), 14965/161, Tehran, Iran; 2 Pediatric Neurology Research Center, Shahid Beheshti University of Medical Sciences, Tehran, Iran; 3 Special Medical Center, Tehran, Iran; 4 Student’s Research Committee, School of Medicine, Shahid Beheshti University of Medical Sciences, Tehran, Iran; 5 Department of Pediatric Endocrinology, Shahid Beheshti University of Medical Sciences, Tehran, Iran; 6 Department of Pediatrics Neurology, Shahid Beheshti University of Medical Sciences, Tehran, Iran; 7 Department of Energy and Environmental Biotechnology, National Institute of Genetic Engineering and Biotechnology (NIGEB), 14965/161, Tehran, Iran; 8 Department of Medical Laboratory Science, Knowledge University, Erbil, Kurdistan Region Iraq

**Keywords:** Niemann-Pick C, Molecular Study, New Mutation

## Abstract

**Background:**

Niemann-Pick disease type C (NPC) is a rare lysosomal neurovisceral storage disease caused by mutations in the NPC 1 (95%) or NPC2 (5%) genes. The products of *NPC1* and *NPC2* genes play considerable roles in glycolipid and cholesterol trafficking, which could consequently lead to NPC disease with variable phenotypes displaying a broad spectrum of symptoms.

**Materials:**

In the present study 35 Iranian NPC unrelated patients were enrolled. These patients were first analysed by the Filipin Staining test of cholesterol deposits in cells for NPC diagnostics. Genomic DNA was extracted from the samples of peripheral blood leukocytes in EDTA following the manufacturer's protocol. All exon–intron boundaries and coding exons of the *NPC1*gene were amplified by polymerase chain reaction (PCR) using appropriate sets of primers. Thereafter, the products of PCR were sequenced and analysed using the NCBI database (https://blast.ncbi.nlm.nih.gov/Blast.cgi). The variants were reviewed by some databases including the Human Gene Mutation Database (HGMD) (http://www.hgmd.cf.ac.uk/ac/index.php) and ClinVar (https://www.ncbi.nlm.nih.gov/clinvar (. Moreover, all the variants were manually classified in terms of the American College of Medical Genetics and Genomics (ACMG) guideline.

**Results:**

The sequence analysis revealed 20 different variations, 10 of which are new, including one nonsense mutation (c.406C > T); three small deletions, (c.3126delC, c.2920_2923delCCTG, and c.2037delG); and six likely pathogenic missense mutations, (c.542C > A, c.1970G > A, c.1993C > G, c.2821 T > C, c.2872C > G, and c.3632 T > A). Finally, the pathogenicity of these new variants was determined using the ACMG guidelines.

**Conclusion:**

The present study aimed to facilitate the prenatal diagnosis of NPC patients in the future. In this regard, we identified 10 novel mutations, and verified that the majority of them occurred in six NPC1 exons (5, 8, 9, 13, 19, and 21), that should be considered with a high priority for Iranian patients' cost-effective evaluation.

## Introduction

Niemann-Pick type C (NPC) disease, is a rare genetic and neurodegenerative disorder induced by intracellular accumulation of free cholesterol and gangliosides into lysosomes or late endosome systems [1]. The NPC was estimated to affect at least one person per 100,000 individuals [2, 3]. Of note, the patients affected by this disease are clinically heterogeneous with a broad spectrum of phenotypes and age of onset is variable. The onset and strictness of illness are determined by the degree of functional disruption in cholesterol trafficking [4]. In the majority of cases, the most common symptoms are neurological and psychiatric symptoms and arise between 4 and 16 years of age [5]. However, the clinical spectrum is ranged from a neonatal fatal disorder to an adult-onset chronic disease. Correspondingly, the neurological symptoms are manifested as mental deterioration, dystonia, dysarthria, dysphagia, ataxia, psychomotor retardation, cataplexy, and various types of seizures usually combined with vertical supranuclear gaze palsy [6, 7]. The neurodegenerative symptoms are often preceded by some visceral complications such as cholestatic jaundice and hepatosplenomegaly [8]. In the NPC patients, the Filipin staining of the cultured fibroblasts has been used extensively as a diagnostic test [9]. The Filipin staining reveals the abnormal intracellular accumulation of cholesterol in fibroblast cells by showing a strong fluorescence in perinuclear vesicles. [10]. Besides, there are several immunologically and ultra-structurally similarities in the brains of patients suffering from Alzheimer's disease and NPC such as the existence of neurofibrillary tangles, endosomal and lysosomal abnormalities [11]. Moreover, the foamy histiocytes (Niemann–Pick cells) could be identified in the bone marrow [8].

NPC is a heterogeneous disorder with two genetic complementation groups [12]. Accordingly, in approximately 95% of NPC patients, mutations are present in the *NPC1* gene (MIM 607623) and the remaining patients have mutations in the *NPC2* gene (MIM 601015) [13]. The *NPC1* gene has 25 exons spanning more than 47 kb in length and is located on chromosome 18q11 [14]. It encodes a mRNA with roughly 4.9-kb that gives origin to a polypeptide with 1,278 amino acids. The NPC protein includes 13 transmembrane domains, six small cytoplasmic loops, three large and four small luminal loops, and one cytoplasmic tail [15, 16]. A cysteine-rich domain (residues 855–1,098) was identified in the third luminal loop [15]. All these functional domains are affected by mutations, which are spread through the *NPC1* gene. Of note most mutations are located on the cysteine-rich domain, including a hot spot region from residues 927 to 958 [17]. Moreover, the region between the amino acid positions 1,038 and 1,253 is known as another hot spot region. This region was shown to have 35% similarity with the Patched 1 (PTC1) protein, namely between residues 974 and 1,180 [18].

The majority of the variations in the *NPC1* gene are missense mutations, small deletions, and insertions [19, 20]. The most important cause of neuronal apoptosis in NPC was recognized to be the accumulation of intracellular free cholesterol in large amounts in the late endosomes or lysosomes, which are caused by a genetic deficit in cholesterol trafficking [21, 22]. However, identification of molecular defects in this disease can be considered as an important confirming diagnostic procedure, allowing a precise and fast prenatal diagnosis. In this study, the analysis of the *NPC1* gene was performed in 35 Iranian patients with NPC, which as a result, led to the detection of 10 new *NPC1* mutations. The present study aimed to provide additional information on the genotype of NPC disease among the Iranian patients.

## Patients and materials

### Patients

We studied 35 Iranian unrelated patients diagnosed as NPC using Filipin staining from 2014 to 2018. Documented consent was obtained from patients as approved for the entire study protocol by the NIGEB ethics committee (IR. NIGEB.EC.1397.8.23. B). Filipin staining of skin fibroblasts was performed in the Centogene GmbH) Rostock, German (. Clinical characteristics and genotype of the NPC patients were summarized in Table 1.

**Table 1:** Clinical and molecular data encountered in 35 NPC patients from Iran

Patient No	Gender	Age	Age of onset	Clinical phenotype	Filipin test	Clinical data						Genotype				Reference
						Dysphagia	Hepatomegaly	VSGP	Splenomegaly	Ataxia	Dementia	DNA change	protein change	Report	Status	
1	M	10	6	J	+	+	Yes	Yes	Yes	Severe	Severe	c.2776G > A	p.Ala926Thr	R	homo	[23]
2	F	8	3	L-I	+	+	Yes	Yes	Yes	Severe	Severe	c.2920-2923delcctg	p.Pro974profsTer8	Not.R	homo	new
3	M	13	10	J	+	+	No	No	Yes	Severe	Severe	c.1970G > A	p.Gly657Asp	Not.R	homo	new
4	M	6	2	E-I	+	+	Yes	Yes	Yes	Severe	Severe	c.1990G > A	P.Val664Met	R	hetero	[18]
												c.2821 T > C	p.Ser941Pro	Not.R	hetero	new
5	F	12	5	L-I	+	+	Yes	Yes	Yes	Severe	Severe	c.506A > T	p.Asn169Ile	R	homo	[24]
6	M	15	10	J	+	+	Yes	Yes	Yes	Severe	Severe	c.1990G > A	P.Val664Met	R	hetero	[18]
												c.3632 T > A	p.Val1211Glu	Not.R	hetero	new
7	M	5.5	1 m*	E-I	+	+	Yes	Yes	Yes	Severe	Severe	c.2821 T > C	p.Ser941Pro	Not.R	hetero	new
												c.2872C > G	p.Arg958Gly	Not.R	hetero	new
8	M	23	11	J	+	+	Yes	Yes	Yes	No	No	c.2821 T > C	p.Ser941Pro	Not.R	hetero	new
												c.2872C > G	p.Arg958Gly	Not.R	hetero	new
9	F	7	3 m	E-I	+	+	Yes	Yes	Yes	Severe	Severe	c.406C > T	p.Gln136Ter	Not.R	homo	new
10	F	12	5	L-I	+	+	Yes	Yes	Yes	No	No	c.2821 T > C	p.Ser941Pro	Not.R	hetero	new
												c.2872C > G	p.Arg958Gly	Not.R	hetero	new
11	M	3	1 m	E-I	+	+	Yes	Yes	Yes	Severe	Severe	c.2776G > A	p.Ala926Thr	R	homo	[23]
12	F	20	9	J	+	+	Yes	Yes	Yes	Severe	Severe	c.542C > A	p.Ala181Asp	Not.R	hetero	new
												c.2821 T > C	p.Ser941Pro	Not.R	hetero	new
13	F	11	4	L-I	+	+	Yes	Yes	Yes	Severe	Severe	c.2821 T > C	p.Ser941Pro	Not.R	hetero	new
												c.2872C > G	p.Arg958Gly	Not.R	hetero	new
14	M	6	6 m	E-I	+	+	Yes	Yes	Yes	Mild	Mild	c.551G > A	p.Cys184Tyr	R	homo	[25]
15	F	22	12	J	+	+	No	Yes	Yes	Mild	Mild	c.1993C > G	p.Leu665Val	Not.R	hetero	new
												c.2821 T > C	p.Ser941Pro	Not.R	hetero	new
16	M	4.5	2 m	E-I	+	+	Yes	Yes	Yes	Severe	Severe	c.3100G > A	p.Gly1034Arg	R	homo	[3]
17	M	9	1	E-I	+	+	Yes	Yes	Yes	Severe	Severe	c.1192C > T	P.His398Tyr	R	homo	[26]
18	M	17	5	L-I	+	+	Yes	Yes	Yes	Severe	Severe	c.2821 T > C	p.Ser941Pro	Not.R	hetero	new
												c.3632 T > A	p.Val1211Glu	Not.R	hetero	new
19	F	25	13	J	+	+	No	Yes	Yes	Mild	Mild	c.2872C > G	p.Arg958Gly	Not.R	hetero	new
												c.3632 T > A	p.Val1211Glu	Not.R	hetero	new
20	F	21	13	J	+	+	Yes	Yes	Yes	Mild	Mild	c.551G > A	p.Cys184Tyr	R	homo	[25]
21	F	11	7	J	+	+	Yes	Yes	Yes	Severe	Severe	c.1990G > A	P.Val664Met	R	hetero	[18]
												c.3632 T > A	p.Val1211Glu	Not.R	hetero	new
22	M	2	2 m	E-I	+	+	Yes	Yes	Yes	No	No	c.1970G > A	p.Gly657Asp	Not.R	homo	new
23	F	16	9	J	+	+	Yes	Yes	Yes	Severe	Severe	c.1970G > A	p.Gly657Asp	Not.R	homo	new
24	F	6	3.5	L-I	+	+	Yes	Yes	Yes	Severe	Severe	c.3126delC	p.His1042GlnfsTer14	Not.R	homo	new
25	F	2.5	7 m	E-I	+	+	Yes	Yes	Yes	Severe	Severe	c.1070C > T	p.Ser357Leu	R	homo	[27]
26	M	3	3 m	E-I	+	+	Yes	Yes	Yes	Severe	Severe	c.3100G > A	p.Gly1034Arg	R	homo	[3]
27	F	17	13	J	+	+	Yes	Yes	Yes	Mild	Mild	c.1415 T > C	p.Leu472Pro	R	homo	[28]
28	M	5	1	E-I	+	+	Yes	Yes	Yes	Severe	Severe	c.3100G > A	p.Gly1034Arg	R	homo	[3]
29	F	7.5	5	L-I	+	+	Yes	Yes	Yes	Severe	Severe	c.1433A > C	p.Asn478Thr	R	homo	[26]
30	F	2	1.5	E-I	+	+	Yes	Yes	Yes	Severe	Severe	c.1433A > C	p.Asn478Thr	R	homo	[26]
31	M	27	10	J	+	+	Yes	No	Yes	No	No	c.1180 T > C	p.Tyr394His	R	homo	[26]
32	M	8	2.5	L-I	+	+	Yes	Yes	Yes	Severe	Severe	c.2037delG	p.Leu680CysfsTer3	Not.R	homo	new
33	M	19	13	J	+	+	No	Yes	Yes	Mild	Mild	c.1192C > T	P.His398Tyr	R	homo	[26]
34	M	12	7	J	+	+	Yes	No	Yes	Severe	Severe	c.1433A > C	p.Asn478Thr	R	homo	[26]
35	F	8.5	4	L-I	+	+	Yes	Yes	Yes	Severe	Severe	c.1415 T > C	p.Leu472Pro	R	homo	[28]

*M* Male, *F* Female, *m* month, *J* Juvenile, *L-I* Late-Infantile, *E-I* Early-Infantile, *VSGP* Vertical supranuclear gaze palsy

*R* Reported, *Not.R* Not Reported

### Blood sampling and DNA extraction

Blood samples were obtained from the Special Medical Center (SMC) and Taban Medical Laboratory, Tehran, IRAN.

Genomic DNA was extracted from the peripheral blood leukocyte samples in EDTA, using the QIA amp kit (QIA amp® DNA Micro Kit #56304, QIAGEN, Hilden, Germany) according with the manufacturer's protocol.

### Polymerase chain reaction (PCR) amplification and sequencing analysis

All 25 coding exons in 24 amplicons and the flanking regions of the *NPC1* gene were amplified by PCR using the primers listed in Table 2. The PCR mixture contained 2 ng DNA template, 20 pmol each primer, 2.5 μL 10 X PCR buffer, and 5 U AmpliTaq in a total volume of 25 μL. The PCR cycle conditions were as following: an initial denaturation at 95°C for 3 min followed by 35 cycles of denaturation at 95°C for 1 min, annealing at 60–63°C for 1 min, and elongation at 72°C for 1 min, with a final incubation for 10 min at 72°C. The PCR amplification products were analysed by 1.5% agarose gel electrophoresis. The PCR products were sequenced using Big Dye Terminator sequencing chemistry (ABI) and the ABI3100 automatic DNA sequence.

**Table 2:** primer sequences for the PCR amplification at the entire coding regions and exon/intron boundaries of the *NPC1* gene

Fragment	Exon	Forward(5’-3’)	Reverse(5’-3’)	Product size(bp)	Tm (°C)
1	E1	AGCCGACGACGCCTTCTTCCTT	ACAAGTGAGGAACCTCCGAGCTC	383	61.6
2	E2	GAAGTTTCTGTGATTGTACTTGAGT	TCCACCTCCACCCTGCAATAACAT	310	61.7
3	E3	GTGTCTTAGTTCACTGAGGAATGTTG	GAAAGCTGAGCATTACCAGTTCACA	253	64.3
4	E4	TGGACACAATAAATGCAGAAAGTAAT	TGACAGGACAACTAAAAGGAACAAT	475	63.4
5	E5	AGCATGGTGCATATGGAGTTCGTG	CAAGCACTGGTGAGCCACTGTGC	369	63
6	E6	GTATTTCAGTGGGCTTTTCTTTGAGT	CATGGAGGTATTTGTTTCTTGTCCTA	475	62.5
7	E7	ACCTCACTGTGATGAAGTCCACTA	CATGACAGACAGCATCATCTGAAC	178	60.7
8	E8	TGATTCCTGCCATGAGATAGCAACT	CCCATCTAGCAGTAGTCAACATGTA	556	60.8
9	E9	ATGTGACGTGTTTCTGGGTTTGC	GTCTTGTTGTTTGCTCACCTCTG	384	62.2
10	E10	AGGTGAGTGCTGAGCTGTATTA	AGGAGATACTATTCTGGGATTCA	403	62.4
11	E11	AGATACAGTCCATAGCTCCAGTGAG	TAAGTGCTTGCTGCAAGTGTCTAGC	288	61.1
12	E12	TCGTGAAAGTTAGGGAGAAGTTT	GGCAACAGAAACGTTACATACAA	312	63.4
13	E13	TTTAGTAACAAGTGGGACAGACAAC	AGGTCACACTCACGAATGCGGAG	339	64.3
14	E14	AGTCCCCCACCGAAGTTTAT	AGCCAGCTCCTTCTTTCTCC	233	61.8
15	E15-16	GCTGTAAACAGAAGTGACGCAGA	CTGGCTTCTTAGAAGGCATGTGAT	480	62.2
16	E17	CCTGTACTCCCTATTAGCCTGTCAT	ACTTGCTTGAAACACCTACGTGCATG	322	63.2
17	E18	TGCTTAGTTACTATCAGAGTGTTCAC	CCTCCTCCGCTGCTTCTGAAGTA	291	61
18	E19	CTGTGGAGCAGGTCAGTAACCCT	GTATAAACTGAGGCACGATGCAAATG	245	62
19	E20	CTTCTAACAGTCCTCCCTGCA	CTGTCTTAGCCCAGTCCTCTC	247	64.3
20	E21	TGCTTAGCCTCAAGTGCTCAGAT	ACCCAGTGTAGGCCCTTTGCTG	337	63.7
21	E22	CATGAGAGGTCAAGTGAGTTG	ATGCTCGCTCCCTCTATG	295	62.2
22	E23	CAGGGTGCCCTGGGTAATTAGCA	GATCCAGACTCTTCAGTCACTGAG	292	61.6
23	E24	TTCAATTACAGGTTGGTAAAAGTGGTT	CTTGAAAAGAATGCCTCAGGATAGA	297	63.1
24	E25	TTCCAAAGTGGGATTACAGGCGTG	GACCGACCCTTAGACACAGTTCAG	221	64.3

The Sequence data were analysed using freely available software (Finch TV) and compared to the query sequence (NM_000271). The variants were reviewed by databases such as ClinVar (https://www.ncbi.nlm.nih.gov/clinvar/) and HGMD (http://www.hgmd.cf.ac.uk/ac/index.php) to determine whether they had been reported previously as pathogenic.

Moreover, all the variants were manually reviewed based on the American College of Medical Genetics and Genomics (ACMG) guideline. Besides, an aggregated knowledge-based tool, VarSome (https: //varsome.com/), was employed to evaluate variants comprehensively.

### In silico analysis

Novel missense variations were analysed using three computational methods, including PolyPhen 2 (http://genetics.bwh.harvard.edu/pph2/), SIFT (http://sift.jcvi.org/), and PANTHER (http://pantherdb.org/about.jsp) to predict the functional impact of novel amino acid changes.

PolyPhen predicts the possible impact of an amino acid substitution on the structure and function of a human protein. The other two in silico approaches were based on evolutionary conservation. Multiple sequence alignments (MSA) in *NPC1* from different species were performed using BoxShade server (version 3.21) to verify the conservation degree.

## Results

Sequence analysis of extracted DNA obtained from the blood samples of 35 patients led to the identification of 20 different variants, 10 of which were previously reported: p.(Asn169Ile), p.(Cys184Tyr), p.(Ala926Thr), p.(Val664Met), p.(Leu472Pro), p.(Asn478Thr), p.(Tyr394His), p.(Gly1034Arg), p.(Ser357Leu), and p.(His398Tyr). These variants were found in the homozygous state in 16 patients (Table 3).

**Table 3:** information about previously reported mutations found in the study

No	Exon No	DNA change	Protein change	Patient No	State	Reference
1	**5**	**c.506A > T**	**p.Asn169Ile**	**5**	**homo**	Reunert (2016) EBioMedicine 4
2	**5**	**c.551G > A**	**p.Cys184Tyr**	**14,20**	**homo**	Chamova (2016) Eur Neurol 75
3	**8**	**c.1070C > T**	**p.Ser357Leu**	**25**	**homo**	Zhonghua Er Ke Za Zhi. 2016
4	**8**	**c.1180 T > C**	**p.Tyr394His**	**31**	**homo**	Tonekaboni*.* Iranian Journal of Child Neurology, 2015
5	**8**	**c.1192C > T**	**p.His398Tyr**	**17,33**	**homo**	Tonekaboni*.* Iranian Journal of Child Neurology, 2015
6	**9**	**c.1415 T > C**	**p.Leu472Pro**	**27,35**	**homo**	ASL SN, Vakili R,*.* Iranian journal of child neurology, 2017
7	**9**	**c.1433A > C**	**p.Asn478Thr**	**29,30,34**	**homo**	Tonekaboni*.* Iranian Journal of Child Neurology, 2015
8	**13**	**c.1990G > A**	**P.Val664Met**	**4,6,21**	**hetero**	Park (2003) Hum Mutant 22, 313
9	**18**	**c.2776G > A**	**p.Ala926Thr**	**1,11**	**homo**	Fernandez-Valero (2005) Clin Genet 68, 245
10	**21**	**c.3100G > A**	**p.Gly1034Arg**	**16,26,28**	**homo**	Yang (2005) J Neurol Neurosurg Psychiatry 76,592

The remaining 10 variations identified include six missense mutations, namely p.(Gly657Asp), p.(Ser941Pro), p.(Arg958Gly), p.(Val1211Glu), p.(Leu665Val), and p.(Ala181Asp), one nonsense mutation p.(Gln136*), and three small deletions, (c.3126delC, c.2920_2923delCCTG, and c.2037delG). None of them have been previously reported. Only five of these 10 variants were found in homozygosity, including the nonsense mutation p. (Gln136*), the missense mutation p. (Gly657Asp), and the three small deletions, (c.3126delC, c.2920_2923delCCTG, and c.2037delG). It is important to notice that in the patients analysed no mutations were observed in any of the intronic flanking regions.

The new variants' pathogenicity, including six likely pathogenic and four pathogenic variants, was determined in terms of the ACMG guidelines.

The predicted functional effects of novel missense variants were determined using the pre-computed values of the SIFT and PANTHER for the tolerated/deleterious effects and Polyphen2 for the benign/damaging effects (Table 4). Stop codon and deletions in the *NPC1* gene were not considered.

**Table 4:** novel variants of *NPC1* gene were detected in 35 Iranian patients and in silico analysis of missense variants (Stop codon and deletions in *NPC1* gene were not considered)

**NO**	**Exon No**	**DNA change**	**Protein change**	**Patient No**	**State**	**SIFT**		**Polyphen**		**PANTHER**	
						**PROVEAN**	**Prediction (cutoff = -2.5)**	**Score**	**Prediction** **(cutoff = 0.5)**	**subPSEC**	**Prediction (cutoff = -3)**
**1**	**4**	**c.406C > T**	**(p.Gln136Ter)**	**9**	**homo**	**-**	**-**	**-**	**-**	**-**	**–**
**2**	**5**	**c.542C > A**	**)p.Ala181Asp(**	**12**	**hetero**	**-4.642**	**Deleterious**	**0.818**	**Probably damaging**	**-2.9001**	**Tolerated**
**3**	**13**	**c.1970G > A**	**)p.Gly657Asp(**	**3,22,23**	**homo**	**-7**	**Deleterious**	**1**	**Probably damaging**	**-5.63539**	**Deleterious**
**4**	**13**	**c.1993C > G**	**)p.Leu665Val(**	**15**	**hetero**	**-2.667**	**Deleterious**	**0.966**	**Probably damaging**	**-3.19574**	**Deleterious**
**5**	**13**	**c.2037delG**	**(p.Leu680CysfsTer3)**	**32**	**homo**	**-**	**-**	**-**	**-**	**-**	**-**
**6**	**19**	**c.2821 T > C**	**)p.Ser941Pro(**	**4,7,8,10,12,13,15,18**	**hetero**	**-4.66**	**Deleterious**	**1**	**Probably damaging**	**-5.14243**	**Deleterious**
**7**	**19**	**c.2872C > G**	**)p.Arg958Gly(**	**7,8,10,13,19**	**hetero**	**-6.191**	**Deleterious**	**0.899**	**Probably damaging**	**-4.0903**	**Deleterious**
**8**	**20**	**c.2920_2923delCCTG**	**)p.pro974profsTer8)**	**2**	**homo**	**-**	**-**	**-**	**-**	**-**	**-**
**9**	**21**	**c.3126delC**	**(p.His1042GlnfsTer14)**	**24**	**homo**	**-**	**-**	**-**	**-**	**-**	**-**
**10**	**24**	**c.3632 T > A**	**)p.Val1211Glu(**	**6, 18,19,21**	**hetero**	**-4.576**	**Deleterious**	**1**	**Probably damaging**	**-4.67659**	**Deleterious**

*PROVEAN* Protein Variation Effect Analyser, *PSEC* position-specific evolutionary conservation

*PANTHER* Protein Analysis Through Evolutionary Relationship, *SIFT* sorting Intolerant From Tolerant

**Fig. 1:**
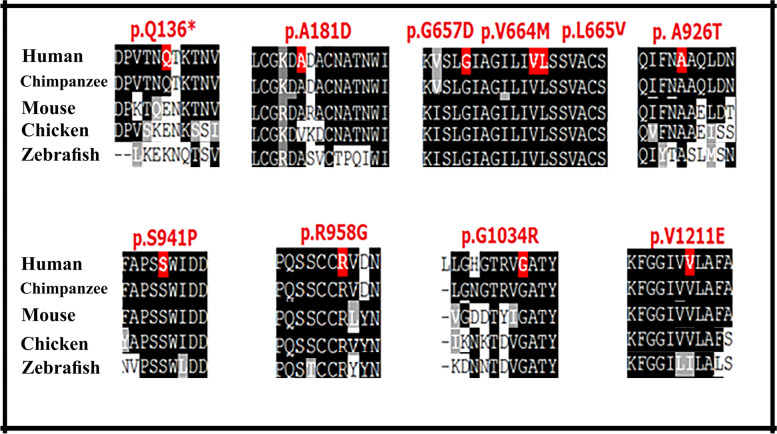
Multiple sequence alignment (MSA) in NPC1 from different species. Conserved amino acid residues and conservative substitutions are highlighted in dark grey and grey, respectively. Amino acid residues in red are affected by novel variants. Human (O15118), chimpanzee (H2QEC5), mouse (O35604), chicken (F1NQT4), and zebrafish (F1QNG7)

MSA of the NPC1 proteins obtained from human (O15118), chimpanzee (H2QEC5), mouse (O35604), chicken (F1NQT4), and zebrafish (F1QNG7) was performed using Clustal Omega and BoxShade server (version 3.21) (Fig. 1).

Observational studies of several national cohorts have categorized patients by age at the onset of neurological manifestations. By considering findings of these studies, the patients were categorized into early-infantile (< 2 years old), late-infantile (2–6 years old), juvenile (6–15 years old), and adult (≥ 15 years old)-onset forms [25]. In this study, the juvenile-onset, early-, and late-infantile onset disease cases were by this order the most frequent disease forms, respectively.

In all patients included in this study, the manifestations usually started in the first decade. The mean age at the time of onset was 5.5 years old (1 m-13y). The disease was diagnosed 2–27 years post the initial clinical presentation. It is noteworthy that variable ages of onset and different age-dependent manifestations, make NPC a complex, complicate, and underdiagnosed disease. Further, dysphagia and splenomegaly were observed in all patients, while hepatomegaly was detected in most patients. Additionally, the neurological features, including vertical supranuclear gaze palsy, cerebellar ataxia, and dementia were highly variable.

## Discussion

The clinical features, age at the clinical symptoms onset, and the rate of neurological symptoms’ progression are highly variable in the NPC disease. In the present study, at the younger age of onset, more severe disease phenotypes were usually observed. Moreover, more than 395 pathogenic variations have been identified for NPC (HGMD Professional). Most of these variations are associated with missense mutations (71%) [16]. A small number of the prevalent variations have been described, including p.(Ile1061Thr) and p.(Pro1007Ala) in the patients from western European descent [29], p.(Arg518Gln) from Japan, and p.(Pro474Leu) from Italy. However, none of these variations was detected in our study [30]. In this work, the c.2821 T > C, p. (Ser941Pro) and c.2872C > G, p. (Arg958Gly) variations, both in exon 19, were the two most common mutations found among NPC patients analysed. Additionally, the majority of the variants were found in exons 5, 8, 9, 13, 19, and 21 (80%) and few mutations have been identified in other NPC exons.

Ten of the twenty variants reported are new variations (not previously reported). Among these, the nonsense mutation and the three small deletions were found highly deleterious.

The new p.(Gln136*) mutation gives origin to a premature termination codon that results in the loss of protein expression and function. Furthermore, the three small deletions were found in the patients p2, p24, and p32 in the homozygous state, and their presence result in frameshifts.

The remaining six new variants were nsSNP (non-synonymous single nucleotide polymorphisms), which were analysed using in silico methods. SIFT and PolyPhen web tools predicted 100% of nsSNPs as "deleterious" and "probable damaging" to protein’s structure and function. By its turn, PANTHER predicted 5 of those 6 nsSNPs as deleterious (83.3%) but one of them (Ala181Asp) was classified as tolerated (Table 4). The variant p. (Gly657Asp) was identified in homozygosity, and the heterozygous state was identified in the *NPC1* gene of their parents.

**Fig. 2:**
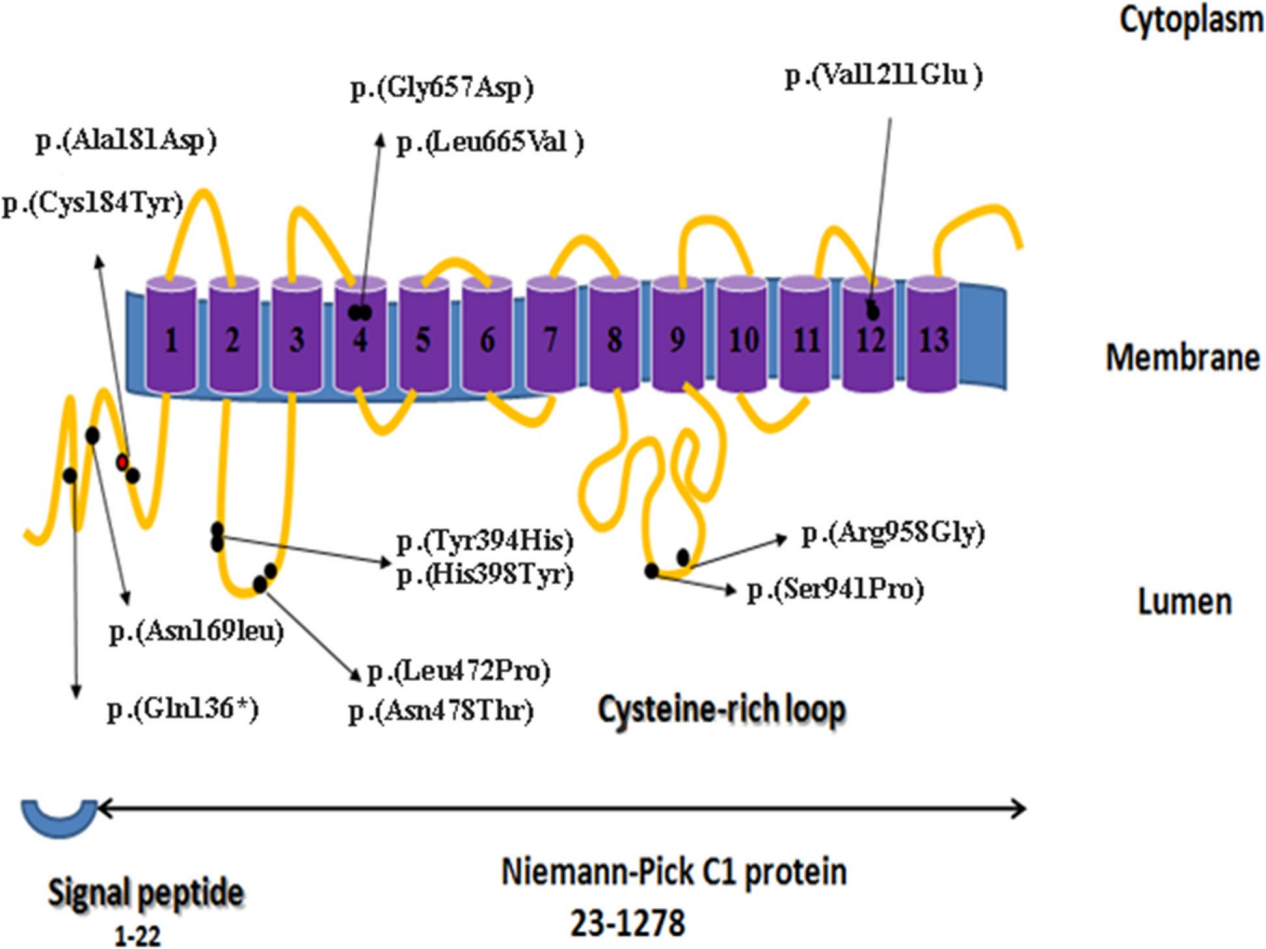
Distribution of the missense mutations found in the present series along the NPC1 protein Domains

The cysteine-rich loop, which is known as a functionally significant protein–protein interaction site, has a ring-finger motif and contains nearly one-third of the *NPC1* variations [31, 32]. In Fig. 2 is presented the location of the detected novel variants on the NPC1 protein structure. It is noteworthy that two of the 10 detected novel variants, including p. (Ser941Pro) and p. (Arg958Gly), were located in the cysteine-rich loop (residues 927–958 a hot spot region of the gene). These variations were found in a heterozygous state in four studied patients, including p7, p8, p13, and p10.

According to findings of our study and the ACMG guideline, some mutations, including p.(Ala181Asp), p.(Gly657Asp), p.(Leu665Val), p.(Ser941Pro), p.(Arg958Gly), and p.(Val1211Glu)are likely pathogenic; these variations according to the ACMG guideline: a) are absent in any frequency database, such as the gnomAD and 1000 Genomes Project, that indicate this mutation is rare (PM2); b) are located in a well-established functional domain (PM1); c) are missense variants in a gene in which benign missense variations are rare so a common mechanism of the disease is considered as the missense variants (PP2), and d) have computational proof supporting a deleterious result on the gene (PP3). Furthermore, p.(Ser941Pro) and p.(Arg958Gly) variants are new missense changes at an amino acid residue, where the other pathogenic missense changes have been seen before (PM5). Based on this evidence, these mutations are classified as likely pathogenic.

In some variations, including p.(Gln136*), c.2037delG, c.2920_2923delCCTG, and c.3126delC: a) the null variants (including frameshift, nonsense, initiation codon, and splice sites) exist in a gene, where LOF (Loss Of Function) is a known mechanism of disease (PVS1); b) the deleterious effect on the gene is supported by computational evidence (PP3).; c) they are absent in all frequency database files such as the gnomAD and 1000 Genomes Project, meaning that these mutations are rare (PM2).

Based on this evidence, these mutations can be classified as pathogenic. In the current study, the aggregated knowledge-based tool, VarSome, was used to review the variants comprehensively.

In this study, 24 out of 35 patients were found to be homozygous, and the remaining patients (11 patients) were either in the heterozygous or in the compounded heterozygous states along with the second mutation.

Seven patients were homozygous, and 11 patients were compound heterozygous for the novel mutations. Furthermore, p. Gly657Asp was detected in three patients (p3-p22-p23), and c.2037delG, c.2920_2923delcctg, c.3126delC, and c.406C > T p.(Gln136*) were detected in the patients. p2, p9, p24, and p32, respectively. In the presence of the all 3 deletions and of the nonsense mutation, severe symptoms were observed.

The new mutations, p.Ala181Asp, p.Ser941Pro, p.Arg958Gly, p.Leu665Val, and p.Val1211Glu were observed in heterozygosity. In all these cases, the manifestations of the disease were early/late infantile and juvenile (p4-p7-p8-p10- p12-p15-p18-p19).

Based on the findings of the present study, no straightforward genotype–phenotype correlations can be established due to the type of new mutations, except in case of the deletions, which presence is always accompanied with severe manifestations of the disease.

The high number of homozygous patients in the present study, could be explained by the high prevalence of consanguineous marriages in Iran and despite patients are not relative they may have common ancestors.

Furthermore, in the present study, some common polymorphisms were found in some of the studied patients, that includes p. (His215Arg) (p6, p12, p21), p. (Ile642Met) (p32), and p. (Ile858Val) (p21). This result is compatible with the previous studies performed in Portugal and Germany [33, 34].

## Conclusions

In conclusion, the mutation screening of 35 Iranian patients with NPC was described, resulting in 10 novel pathogenic and likely pathogenic *NPC1* gene variants. The Niemann-Pick is a neurological disorder with a broad spectrum of clinical features. An alternative tool used to confirm the diagnosis of NPC was mutation screening. Finally, the detection of mutations will facilitate carrier screening of family members and prenatal diagnosis.

## Data Availability

The variants in this article are available in National Center for Biotechnology Information (NCBI) and the datasets used and analysed during the current study are available from the corresponding author on reasonable request. NCBI database:(https://blast.ncbi.nlm.nih.gov/Blast.cgi) The Human Gene Mutation Database: http://www.hgmd.cf.ac.uk/ac/all.php. ClinVar: https://www.ncbi.nlm.nih.gov/clinvar/. VarSome: https://varsome.com/. UniProt: https://www.uniprot.org/.

## Abbreviations

NPC: Niemann-Pick C
PCR: Polymerase chain reaction
NCBI: National Center for Biotechnology Information
NIGEB: National Institute of Genetic Engineering and Biotechnology
PROVEAN: Protein Variation Effect Analyser
PSEC: Position-specific evolutionary conservation
PANTHER: Protein Analysis Through Evolutionary Relationship
SIFT: Sorting Intolerant From Tolerant
HMG-R: Hydroxyl methyl glutaryl coenzyme A reductase
SMC: Special Medical Center
nsSNP: Non-synonymous single nucleotide polymorphisms
